# Cerebral HSV-1 Vasculitis as a Fatal Complication of Immunosuppression in Non-Hodgkin´s Lymphoma: A Case Report and Review of the Literature

**DOI:** 10.3390/pathogens9030193

**Published:** 2020-03-05

**Authors:** Raffaele Nardone, Luca Carnicelli, Francesco Brigo, Slaven Pikija, Larissa Hauer, Johann Sellner

**Affiliations:** 1 Department of Neurology, Franz Tappeiner Hospital, 39012 Merano, Italy; raffale.nardone@gmail.com (R.N.); luca.carnicelli@sabes.it (L.C.); dr.francescobrigo@gmail.com (F.B.); 2 Department of Neurology, Christian Doppler Medical Center, Paracelsus University Salzburg, 5020 Salzburg, Austria; s.pikija@salk.at; 3 Department of Psychiatry, Psychotherapy and Psychosomatic Medicine, Christian Doppler Medical Center, Paracelsus University Salzburg, 5020 Salzburg, Austria; l.sellner@salk.at; 4 Department of Neurology, Klinikum rechts der Isar, Technische Universität München, 81675 München, Germany; 5 Department of Neurology, Krankenhaus Mistelbach-Gänserndorf, 2130 Mistelbach, Austria

**Keywords:** viral encephalitis, cerebral vasculitis, opportunistic infection, splenic marginal cell lymphoma, Non-Hodgkin´s lymphoma, immunosuppression

## Abstract

Patients with lymphoma are predisposed to infection because of the immunocompromised state related to the disease itself and as a consequence of chemo-/radiotherapy. Here, we report a case of Herpes-simplex virus encephalitis (HSE) in an immunosuppressed patient with splenic marginal zone lymphoma (SMZL), a rare indolent variant of non-Hodgkin´s lymphoma (NHL). The course was complicated febrile neutropenia and HSV-1-related cerebral vasculitis causing progressive ischemic stroke. This case illustrates the expanding spectrum of atypical clinical and radiological manifestations of HSE in patients treated with myelotoxic drugs. Moreover, we summarize the few central nervous system manifestations of SMZL reported in the literature and discuss distinct causes of neurological deterioration in patients with NHL.

## 1. Introduction

Splenic marginal zone lymphoma (SMZL) is a rare indolent non-Hodgkin lymphoma (NHL) subtype that originates from B memory lymphocytes present in the marginal zone of secondary lymphoid follicles [1,2]. The median age at diagnosis of SMZL is 69 years and there is an association with hepatitis C infection in Southern Europe [3]. Patients usually present massive splenomegaly and bone marrow involvement with minimal or absent lymphadenopathy except for the spleen hilum [4]. Extranodal involvement incudes the bone marrow and the liver, whereas primary or secondary central nervous system (CNS) manifestations are extremely rare [5]. Of note, patients with lymphoma are predisposed to infection because of the immunosuppressed state related to the disease itself and chemo-/radiotherapy [6]. 

While the epidemiology of viral encephalitis is in constant turnover, Herpes-simplex virus (HSV) remains the most common pathogen of sporadic encephalitis (HSE) worldwide [7]. HSE is not regarded as an opportunistic infection. Notably, there is an increasing number of reports about HSE in immunosuppressed patients and treatment with immunotherapies approved for disease modification in multiple sclerosis (MS) is expanding [8,9,10,11]. HSE in patients on immunosuppressive treatment is associated with atypical clinical and radiological presentation and poorer outcome [12]. In this regard, prodromal symptoms such as fever and headache are less frequent in immunosuppressed patients and there is a higher chance of additional extratemporal location of lesions in the brainstem and the cerebellum or widespread cortical involvement. Moreover, an increasing number of patients have been reported to develop this condition following brain radiation or neurosurgical intervention, respectively [13,14]. 

Here, we describe the case of HSE with cerebral vasculitis causing progressive ischemic stroke in an immunosuppressed patient with SMZL. 

## 2. Case Study

**Figure 1:**
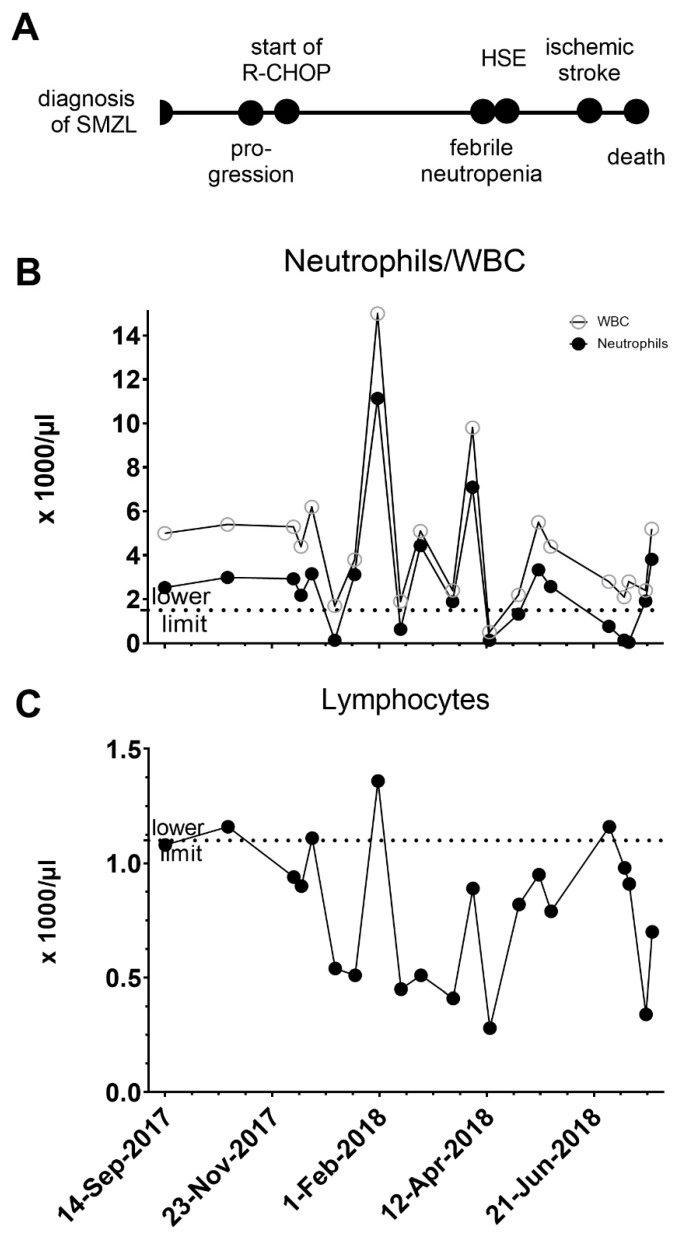
(**A**) Time course of clinical events from diagnosis of splenic marginal zone lymphoma (SMZL) to febrile lymphopenia and Herpes-simplex virus encephalitis (HSE). (**B**,**C**) Temporary profile of white blood cells (WBC), neutrophils and lymphocytes. The lower limit for neutrophils and lymphocytes is depicted by a dotted horizontal line (1.5 × 1000 cells/µL and 1.1 × 1000 cells/µL)).

A 60 year old male patient was diagnosed with SMZL, an indolent B-cell NHL, stage IVB. The presenting symptoms included abdominal pain, fatigue and fever. His medical history included hypertension, hypercholesterinemia and ischemic cardiomyopathy. The CT scans showed splenomegaly (14 × 9 cm) and enlarged retroperitoneal lymph nodes (up to 15 mm). Bone marrow examination detected findings supportive of SMZL including CD20-positive malignant B cells and a lack of CD5 and CD30 expression. Two weeks before the scheduled therapy with R-CHOP (rituximab, cyclophosphamide, hydroxydaunorubicin, vincristin, prednisone), he was admitted with fever and abdominal pain. The work-up revealed progress of splenomegaly (17 × 11 cm) and new development of intrasplenic lesions consistent with splenic infiltration by the lymphoma. He was anemic and received erythrocyte concentrates. A radiation therapy was not performed. He then received treatment with R-CHOP and filgrastim, a biosimilar of Granulocyte colony-stimulating factor (G-CSF) with each course as shown in Figure 1. The time course of leukocyte dynamics is shown in Figure 1, and the nadir of the neutrophils was 130, 630 and 180 cell/µL, respectively. He was not lymphopenic the days before the start of R-CHOP therapy but had almost continuously grade 2-3 lymphopenia thereafter according to the Common Terminology Criteria for Adverse Events (CTAE) Grading system. 

Shortly after the fourth R-CHOP cycle he sought medical help for nausea and emesis. He was diagnosed febrile neutropenia (grade 4 (130 cells/µL)). He developed sinusitis and persistent and high fever despite prophylaxis with broad-spectrum antibiotic and antifungal therapy. He was treated with 2 × 30 Mio U. filgrastim. His clinical condition further deteriorated and he reported headache and had recurrent episodes of reduced vigilance and confusion. In addition, epileptic seizures were observed. While brain magnetic resonance imaging (MRI) was unremarkable and electroencephalography (EEG) ruled out status epilepticus. The CSF examination revealed a lymphomononuclear pleocytosis of 230 cells/µL and increased blood-brain barrier (BBB) permeability (protein in CSF 203 mg/dL, upper limit 45 mg/dL). Empirical acyclovir (10 mg/kg iv every 8 hr) was started on suspicion for HSE, and later HSV-1 was confirmed by polymerase chain reaction (PCR) of CSF specimen. He fulfilled the diagnostic criteria for confirmed encephalitis [15]. The clinical condition improved under anticonvulsive and antiviral therapy, and the latter was given for 21 days. The hospital stay was 5 weeks, the last neutrophil count was 1320 cells/µL. 

Eight weeks later he was readmitted for increasing confusion. He was still neutropenic (770 neutrophils/µL). CSF examination revealed a persistent mononuclear pleocytosis (19 cells/µL), BBB disruption and positive PCR for HSV-1. We performed another course of IV acyclovir for 14 days. Again, PCR was negative for common causes of opportunistic infections including cytomegalovirus (CMV), varicella-zoster virus (VZV), EBV and toxoplasmosis. The onconeural antibodies (anti-Hu, Yo, Ri, Ma, CV2 and amphiphysin), and autoimmune (anti-nuclear, -cardiolipin, ds-DNA, myeloperoxidase) as well as anti-N-methyl-D-aspartate receptor (NMDAR) antibodies were negative. 

**Figure 2:**
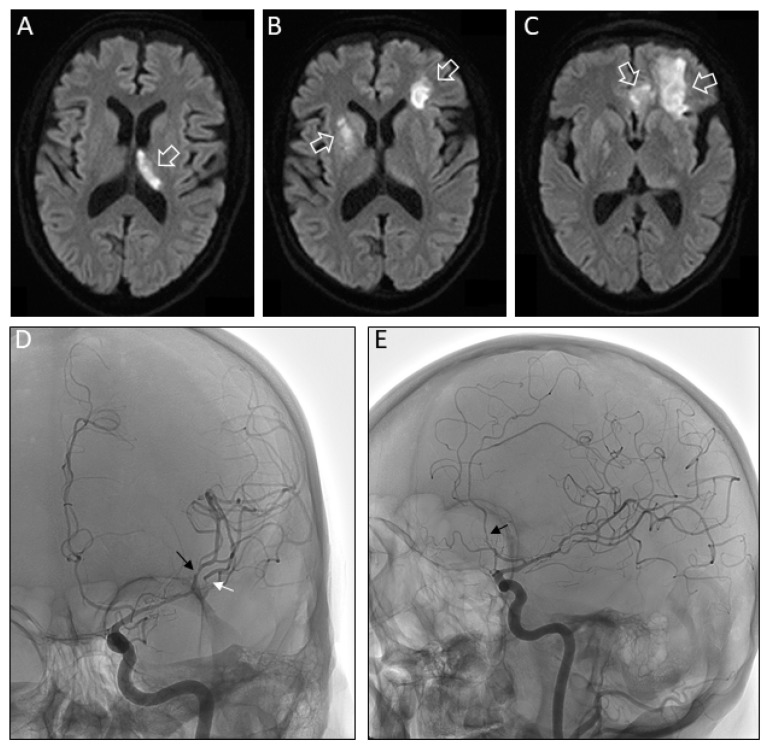
(**A**) Fluid attenuated inversion recovery (FLAIR) MR axial images of the brain on second admission reveals acute infarction in the region of the left thalamus (white open arrow). (**B**) Seven days later additional FLAIR hyperintensities consistent with brain infarction were present in the region of the right putamen and in left juxtacortical frontal lobe (white open arrows). (**C**) The previous infarction also extended further rostral and affected white matter and U-fibers in the left frontal lobe and a portion of the left cingulate gyrus (white open arrows). (**D**) Anterior-posterior projection of early arterial phase of digital subtraction angiography (DSA) after selective left internal carotid catheterization showing multifocal segmental narrowing in the left superior (black arrow) and inferior (white arrow) M2 segments of the middle cerebral artery. Lateral DSA projection showing narrowing in left anterior cerebral artery (Panel **E**).

Brain magnetic resonance imaging (MRI) disclosed a recent infarction of the left thalamus (Figure 2A). We observed new neurological symptoms over the next days with weakness and numbness on the left side, fluctuating cognitive function, mood and behavior. Brain MRI on day 7 from readmission showed further new ischemic lesions in the previously affected as well as additional vascular territories (Figure 2B,C). Conventional angiography revealed narrowing of intracerebral cerebral vessels consistent with vasculitis. The abnormalities were most prominent in the M2 segments of the left medial and the anterior cerebral artery (Figure 2D,E). High dose methylprednisolone (1g per day) was added for 7 days and acetylsalicylic acid switched to clopidogrel. There was no evidence for cardiac arrhythmia on Holter-ECG or cardiac thrombus or endocarditis on echocardiography. Yet, clinical deterioration was not halted and the patient died one month later in a palliative care setting. The approval by the local ethics committee is on file (415-EP/73/748-2017). 

## 3. Discussion

Opportunistic CNS infections play a significant role in life-threatening chemotherapy- and radiation-related complications. Since the CSF cell count can be normal in immunocompromised individuals and the clinical presentation may be distinct, antiviral treatment on suspicion is commonly delayed [16]. Of note, immunocompromised patients have six times higher chance for an unfavorable outcome than patients with an unaltered immune system [12]. Myelotoxicity is a life-threatening complication of chemotherapy with R-CHOP. The risk for febrile neutropenia is 20% and the additional use of G-CSF was shown to allow the administration of maximal relative dose intensities [17]. Infections with Herpes-simplex and -zoster viruses are known complications of R-CHOP for treatment of B-cell lymphoma [18]. This case expands the spectrum as cerebral vasculitis in HSE, which is almost exclusively related to type 2 virus [19]. Epileptic seizures and ischemic stroke are risk factors for poor prognosis in HSE and presented in our patient [20]. The likelihood for an unfavorable outcome in patients with HSE is also higher with acute respiratory failure and intracranial hemorrhage. 

Cerebral vasculitis or vasculopathies have not been described as a CNS manifestation or complication of SMZL Several studies corroborated that the risk of CNS involvement for indolent lymphomas was low. In a study of 1163 patients with low-grade non-Hodgkin’s lymphoma, the rate of patients with CNS involvement at 5 years was 2.8% [21]. Of note, CNS involvement has been reported in 7% of patients with low-grade non-Hodgkin’s lymphoma, which was associated with a transformation to high-grade malignancy in all these patients [22]. Such a biopsy-confirmed case was reported by Gao [23], where the secondary cerebral manifestation presented on MRI as a tumorous brain mass (2.1 × 2.2 cm). That radiological presentation contrasts our case with the presence of vascular alterations. The additional option beyond brain biopsy to verify a potential neoplastic origin of the cerebral lesion is positron emission tomography (PET) imaging, which was not performed in our patient. A patient with SMZL who developed focal neurological symptoms 3.5 years after diagnosis of the lymphoma was reported by Toennissen et al. [24]. MRI revealed four tumorous lesions and strong uptake on [18F] fluorodeoxyglucose (FDG) PET. The biopsy confirmed transformation to a diffuse large B-cell lymphoma and secondary CNS manifestation. Fadugba [25] reported a case of cerebral manifestation of SMZL, which was a cerebral mass with a 4-cm diameter and a large amount of cerebral edema, resulting in a midline shift. Wedgewood et al. [26] reported a patient with recurrence of SMCL as intracerebral dural mass. These dural manifestations may resemble a meningioma. Moreover, there are a few cases with clinical presentations related to meningeal lymphomatosis [27,28,29,30]. In our patient, cytology did not identify atypical cells in the CSF and there was no meningeal enhancement on MRI, which is frequently seen in the case of meningeal lymphomatosis. There are anecdotal reports about the efficacy of intrathecal treatment for these patients [31]. 

Persistent lymphopenia or marked drop of lymphocytes is a known risk factor for opportunistic CNS infection [32]. Of note, HSE has been reported not only after immunosuppressive therapy but also in multiple sclerosis (MS) by lymphocyte-lowering immunotherapies or interference with the migratory action of lymphocytes [10,33,34]. Neutrophils have an essential biological function for both innate and adaptive immune responses. Our patient had chemotherapy-induced febrile neutropenia and persistent grade 2-3 lymphopenia, which both are likely to have contributed to the development of HSE. Neutrophils play a role in the first line of host defense and are present in CSF in the early course of HSE [35]. While their exact role in the immunopathogenesis of HSE is unclear, neutrophils were critical for the control of replication and spread of the virus in an murine model of HSV-1 keratitis [36]. This effect is likely to be mediated by the release of oxygen and nitrogen reactive species as well as TNF-α. The latter activates caspase 8-dependent pathways, which can induce cell lysis and apoptosis of viral-infected epithelial cells [37]. 

Uncomplicated HSE is treated for 2 weeks with intravenous acyclovir. In contrast, guidelines recommend intravenous acyclovir for 3 weeks in immunocompromised patients and a retap thereafter to exclude persistent infection [38]. In this regard, there is ongoing research to assess whether HSV strains in immunodeficient patients are more prone to developing acyclovir resistance [39]. With the development of ischemic stroke in territories supplied by distinct cerebral arteries in febrile neutropenia, we needed to rule out systemic, thoracic and abdominal infectious foci, endocarditis and cardiac arrhythmia as a potential embolic source. While his clinical presentation and subacute and fluctuating course was encephalitis-like and CSF consistent with intrathecal inflammation, we could not detect encephalitic lesions on conventional imaging during the initial stage of neurological symptoms. Of note, the mesial temporal lobe is the predilection site for brain lesions in HSE but an additional extratemporal location is more frequent in immunosuppressed patients [12]. The focal lesions seen in our patient, however, are of an ischemic nature and a consequence of widespread vasculitis of cerebral vessels. Infarction in infectious cerebral vasculitis can not only occur due to reduced cerebral blood flow caused by inflammation of the vessel wall but also vasospasm and arterio-arterial thrombosis [40]. Favorable effects by steroids may be particularly seen in patients with HSE-related cerebral vasculitis. As steroids have anti-inflammatory effects and could thus promote viral replication, some centers reserve this treatment for patients with brain edema and mass effect [41]. Moreover, there is no consensus regarding the steroid preparation and dosage, and some use a 4-day course of dexamethasone and others up to 7 days of methylprednisone. 

However, in addition to secondary CNS manifestation and HSV-1 related vasculitis, there are further causes of neurological deterioration in SMZL. Posterior leukoencephalopathy syndrome and vasoconstriction syndrome are clinical mimics of encephalitis, that show vascular alterations and has been reported in the context of lymphoma and with the oncological therapies used in our patient [42,43]. Ibrutinib, a Bruton’s tyrosine kinase inhibitor, has been increasingly widely used in relapsed and refractory SMZL. Cases of cryptococcal meningoencephalitis were confirmed for patients on this treatment [44]. In addition, in patients with myelotoxic adverse events, the entire range of opportunistic brain infection needs to be taken into account.

## 4. Conclusions

There are various conditions which can cause neurological deterioration in patients with SMZL. The spectrum includes potentially reversible conditions such as encephalopathies and vasculopathies but also disorders with high mortality and morbidity including malignant transformation with secondary CNS manifestation and opportunistic infections. We described the rare case of HSV-1-related cerebral vasculitis causing progressive ischemic stroke, which developed as a complication of myelotoxicity by chemotherapy.
